# 

**Published:** 1888-12

**Authors:** 


### CONTENTS—DECEMBER, 1888.—VOL. IV., NO. 6.

ORIGINAL ARTICLES— Page.
The Appearance and Behavior of the Pupil in Health and Disease. By T. J. Tyner, M. D., Austin, Texas. 237
Apoplexy. By Jno. Preston, M. D., Austin, Texas . . 241
A Successful Vaginal Hysterectomy for Carcinoma Uteri. By W. H. Wathen, M. D., Louisville, Ky . 248
Dermoid Cysts of the Coccygeal Region. By E. J. Beall, M. D., Ft. Worth, Texas . . . . . . . . . . 254

CORRESPONDENCE—
The Hog as an Article of Food. Letter from Dr. Cunningham, M. D., Dallas, Texas . . . . . . . . . . 260
Letter from Dr. Stovall, of Terrell . . . . . . . . . . 262

SOCIETY NOTES—
The Southern Surgical and Gynecological Association. Proceedings of Late Meeting . . . . . . . . . . . . . 264
In Memoriam. Resolutions by B. and M. Medical Society on the Death of Dr. E. Mellou . . . . . . . . . 267

EDITORIAL—
Physicians' Mutual Benefit Association of Texas. Officers and organization . . . . . . . . . . . . . . . . 269
Circular Letter to the Physicians of Texas, by the Board of Directors P. M. B. A . . . . . . . . . . . 270
"It Smells to Heaven." . . . . . . . . . . . . . . . 273
Dr. Cunningham's Defense of the Hog . . . . . . . . 276

MEDICAL NEWS AND MISCELLANY—
Death of Dr. Baker, Marriages and Deaths, Assassination of Dr. Beidler, Practice for Sale, etc . . . . . . 277
PUBLISHERS NOTES— . . . . . . . . . . . . . . . . . . . . 280

